# Oral supplementations of betaine, choline, creatine and vitamin B_6_ and their influence on the development of homocysteinaemia in neonatal piglets

**DOI:** 10.1017/jns.2015.19

**Published:** 2015-09-22

**Authors:** Marie-Édith Côté-Robitaille, Christiane L. Girard, Frédéric Guay, J. Jacques Matte

**Affiliations:** 1 Dairy and Swine Research and Development Centre, Agriculture and Agri-Food Canada, 2000 College Street, Sherbrooke, QC, CanadaJ1M 0C8; 2 Faculté des sciences de l'agriculture et de l'alimentation, Département de sciences animales, Université Laval, Québec, QC, CanadaG1V 0A6

**Keywords:** Homocysteine, Nutrient regulators, Neonatal nutrition, Piglets, Hcy, homocysteine, pHcy, plasma homocysteine, P-5-P, pyridoxal-5-phosphate

## Abstract

Homocysteine (Hcy) is an intermediary sulphur amino acid recognised for pro-oxidative properties in several species which may weaken immune competence in piglets. In this species, there is an acute 10-fold increase of concentrations of plasma Hcy (pHcy) during the first 2 weeks of life. The present experiment aimed to determine if pHcy in piglets can be regulated by oral supplementations of betaine as a methyl group supplier, creatine for reducing the demand for methyl groups, choline with both previous functions and vitamin B_6_ as enzymic co-factor for Hcy catabolism. A total of seventeen sows (second parity) were fed gestation and lactation diets supplemented with folic acid (10 mg/kg) and vitamin B_12_ (150 µg/kg). Eight piglets in each litter received daily one of the eight following oral treatments (mg/kg body weight): (1) control (saline); (2) betaine (50); (3) choline (70); (4) creatine (300); (5) pyridoxine (0·2); (6) treatments 2 and 5; (7) treatments 3 and 4; and (8) treatments 2, 3, 4 and 5. According to age, pHcy increased sharply from 2·48 µm at birth to 17·96 µm at 21 d of age (*P* < 0·01). Concentrations of pHcy tended to be lower (*P* = 0·09) in treated than in control piglets but the highest and sole pairwise significant decrease (23 %) was observed between treatments 1 and 8 (*P* = 0·03). Growth from birth to 21 d of age was not influenced by treatments (*P* > 0·70). Therefore, it appears possible to reduce pHcy concentrations in suckling piglets but a combination of all chosen nutrients is required.

Homocysteine (Hcy) is a sulphur-containing amino acid derived from *S*-adenosyl-Hcy produced from *S*-adenosyl-methionine, the major cellular methyl donor. Hcy is known as a powerful pro-oxidant^(^^1^^)^ with multiple deleterious effects notably on the immune system in humans^(^^2^^)^. In suckling piglets, there is a rapid postnatal development of homocysteinaemia^(^^3^^,^^4^^)^. This condition is potentially harmful for these young animals as a model comparing two populations of piglets with high (24·7 µm) or low (16·7 µm) homocysteinaemia showed that indicators of cell-mediated immunity (proliferation of lymphocytes in response to the mitogen concanavalin A) were negatively affected in the former group of piglets, suggesting a weakened immune competence associated with high plasma concentrations of Hcy^(^^5^^)^.

Blood plasma total Hcy (pHcy), the most common indicator of Hcy status, decreases in sows and piglets following administration of dietary supplements of folic acid and vitamin B_12_ to sows during gestation^(^^4^^,^^6^^,^^7^^)^ and lactation^(^^5^^)^. These responses in sows and piglets were associated with remethylation where vitamin B_12_-dependent methionine synthase transfers a methyl group from CH_3_-H_4_-folate to convert Hcy in methionine^(^^8^^)^. Nevertheless, the lower concentrations of pHcy in these suckling piglets remain two to five times higher than in other species^(^^4^^,^^5^^)^. Therefore, other metabolic pathways may be involved for Hcy homeostasis in these animals.

The demand for methyl groups which frequently results in accumulation of Hcy is particularly high in piglets for *de novo* synthesis of creatine^(^^9^^)^. The same situation applies for endogenous phosphatidylcholine and choline^(^^10^^)^. Supplements of these preformed metabolites could reduce the need for methyl groups and prevent accumulation of Hcy. Besides the remethylation pathway via methionine synthase, the disposal of Hcy may also proceed through another remethylation pathway via betaine-Hcy-methyltransferase. Last, Hcy can be catabolised by the trans-sulfuration pathway where the vitamin B_6_-dependent cystathionine β-synthase and cystathionine-*γ*-lyase produce cystathionine and then cysteine and glutathione^(^^8^^)^.

Therefore, it was hypothesised that pHcy in piglets can be regulated by exogenous oral supplements of betaine as a methyl group supplier, creatine for reducing the demand for methyl groups, choline as methyl group supplier and/or for reducing the demand for methyl groups and vitamin B_6_ for covering co-enzyme needs for Hcy catabolism.

## Experimental methods

The experimental procedures followed the guidelines of the Canadian Council on Animal Care^(^^11^^)^ and were approved by the Institutional Animal Care Committee of the Dairy and Swine Research and Development Centre of Sherbrooke (QC, Canada). All animals were cared for and slaughtered according to the recommended code of practice of Agriculture Canada^(^^12^^)^.

### Sow management

A total of seventeen Yorkshire–Landrace sows (second parity) were used for this experiment. The average body weights at insemination, farrowing and weaning were 205·8, 274·1 and 244·0 (sem < 7·0) kg, respectively. From 3 d after weaning, oestrus was detected twice per d when a boar was introduced near the pen for 1 h between 08.00 and 09.00 hours and from 16.00 to 17.00 hours. When oestrus was first detected in the morning, sows were inseminated twice, 8 and 24 h following detection, whereas when oestrus was first detected in the afternoon, the two inseminations were done 16 and 24 h later. Sows were artificially inseminated with 85 ml of semen (2 × 10^9^ live sperm cells from pooled semen of three Duroc boars) provided by a local artificial insemination centre (Centre d'Insémination Porcine du Québec Inc., St-Lambert, QC, Canada). During gestation, sows were kept in individual stalls and individually fed 2·5 kg of a diet supplemented with folic acid (10 mg/kg) and vitamin B_12_ (150 µg/kg) (Table 1). These levels of dietary supplementation were chosen according to Simard *et al.*^(^^4^^)^ and Guay *et al.*^(^^7^^)^. At 1 week before expected farrowing, sows were transferred from the gestation (0·6 × 2·2 m, half-slatted concrete flooring) to the farrowing crate (1·5 × 2·2 m, cast iron and plastic-coated wire mesh flooring). The farrowing–lactating room housed five sows and was maintained at a temperature of 24°C. At 113 d of gestation, sows received an intravulvar infusion of 1·0 ml of dinoprost tromethamine (Lutalyse; Pfizer Animal Health) to induce delivery. After 24  h, if sows had not delivered, they received an intramuscular (neck) injection of 0·5 m of synthetic oxytocin (20 IU/ml, P.V.U., Oxyto-Sure; Vétoquinol). During lactation, the diet was also supplemented with folic acid and vitamin B_12_ at the same concentrations as for the gestation diet (Table 1). The feed allowance was 3·25 kg on day 1 and was increased by 0·5 kg/d until it reached *ad libitum* intake at the end of first week of lactation. Measures of body weight and back fat thickness were recorded at mating, on day 1 after farrowing and at weaning. Blood samples were taken when sows were transferred in the farrowing crate, on day 1 of lactation and at weaning. Milk samples were collected on days 1 and 21 of lactation as described by Simard *et al.*^(^^4^^)^ and Audet *et al.*^(^^5^^)^.

**Table 1.** Composition of the basal diet (as-fed basis) for gestation and lactation periods

Ingredient	Gestation (%)*	Lactation (%)†
Maize	56·29	69·36
Red wheat middlings	20·00	
Soyabean hulls	8·60	
Rapeseed meal	8·00	2·50
Soyabean meal (48 %)	3·40	23·90
Limestone	1·94	1·99
Monocalcium phosphate	0·65	1·11
Salt	0·61	0·53
Mineral and vitamin premix	0·20‡	0·20§
Choline chloride	0·10	0·10
Phytase premix	0·04	0·08
l-Lysine	0·06	0·12
Mold inhibitor||	0·10	0·10
Vitamin B_12_ premix¶	0·01	0·01

* The proximate analysis for crude protein, crude fat, crude fibre, Ca and P of the basal diet was 13·5, 3·8, 4·6, 1·2 and 0·7 %, respectively. Metabolisable energy was estimated at 12·2 MJ/kg. The calculated lysine, and lysine:methionine and lysine:methionine + cysteine ratios were 0·62 %, 2·58 and 1·19, respectively.

† The proximate analysis for crude protein, crude fat, crude fibre, Ca and P of the basal diet was 17·9, 3·2, 2·5, 1·0, and 0·6 %, respectively. Metabolisable energy was estimated at 12·3 MJ/kg. The calculated lysine, and lysine:methionine and lysine:methionine + cysteine ratios were 0·97 %, 3·34 and 1·62, respectively.

‡ Supplied (per kg of feed): Mn, 86·2 mg; Zn, 188·5 mg; Fe, 345·8 mg; Cu, 28·5 mg; I, 2·1 mg; Se, 0·3 mg; vitamin A, 14 564 IU; vitamin D, 1500 IU; vitamin E, 60 IU; vitamin K, 2·6 mg; vitamin B_12_, 0·03 mg; thiamine, 2·7 mg; riboflavin, 5·0 mg; niacin, 31·1 mg; pantothenic acid, 21·3 mg; folic acid, 10 mg; pyridoxine, 2·6 mg; biotin, 0·4 mg; choline, 520·7 mg.

§ Supplied (per kg of feed): Mn, 66·8 mg; Zn, 180·9 mg; Fe, 347·4 mg; Cu, 28·7 mg; I, 2·1 mg; Se, 0·3 mg; vitamin A, 14 564 IU; vitamin D, 1500 IU; vitamin E, 60 IU; vitamin K, 2·6 mg; vitamin B_12_, 0·03 mg; thiamine, 2·7 mg; riboflavin, 5·0 mg; niacin, 31·1 mg; pantothenic acid, 21·3 mg; folic acid, 10 mg; pyridoxine, 2·6 mg; biotin, 0·4 mg; choline, 520·7 mg.

|| Myco Curb^®^, Kemin Industries Inc.

¶ This premix was given as a top dressing which provided an equivalent of 116·4 µg/kg of vitamin B_12_ (analysed according to Girard *et al*.^(^^32^^)^).

### Piglets and treatments

During farrowing, all piglets were placed in a box outside the crate to prevent access to mammary glands and colostrum intake until body weight measurement and a first blood sample were taken (day 0, within 1 h after birth). Teeth were cut and an Fe intramuscular injection (1 ml of iron dextran containing 100 mg of Fe/ml, Ironol, P.V.U., Lavaltrie) was given on day 1. A total of eight piglets of average body weight and condition (visual assessment) were chosen and assigned randomly to their respective treatments while the four remaining piglets did not receive any treatment but were kept with their dams in order to maintain the litter size at twelve piglets. On day 1, when needed to reach a litter size of twelve piglets, piglets (*n* ≤ 4) were fostered but those animals were not treated. Eight different treatments were compared within each litter: (1) control (0·9 % NaCl); (2) betaine; (3) choline; (4) creatine; (5) vitamin B_6_; (6) betaine and vitamin B_6_; (7) choline and creatine; and (8) betaine, vitamin B_6_, choline and creatine. Each treatment consisted of commercially available forms of these nutrients and was given as a liquid oral bolus, every day at the same time (13.00 hours) from day 0 until day 21 (weaning). Piglets were weighed and blood samples were collected from the jugular vein by venepuncture^(^^13^^)^, on day 0 and then before the administration of treatments on days 1, 7, 14 and 21 of lactation. Before weighing and blood sample collection, all piglets were separated from their mother for 1 h. This period, which is slightly longer than the normal natural interval between two suckling episodes^(^^14^^)^, allowed us to standardise for the effect of previous milk intake on body weight and plasma metabolite concentrations.

### Solutions for oral administration

Solutions were prepared with betaine hydrochloride (B7045, Sigma-Aldrich), choline chloride (70 % liquid; Chinook, Nutreco) and pyridoxine hydrochloride (P9755; Sigma-Aldrich). For each nutrient, stock solutions were prepared in water and frozen at −20°C. These stock solutions were further diluted in water as working solutions providing the target daily amounts for each nutrient (Table 2). For creatine treatments, creatine monohydrate (C3630; Sigma-Aldrich) was used and as the solubilisation was not complete, the daily allowance was weighed in a tube and diluted directly in water as a working solution for treatment 4 or within the appropriate working solutions for treatments 7 and 8. Daily allowances of betaine, choline, creatine and pyridoxine were adjusted every week based on intakes per kg body weight derived from studies of Eklund *et al.*^(^^15^^)^, Brosnan *et al.*^(^^16^^)^, Donovan *et al.*^(^^17^^)^ and Matte *et al.*^(^^18^^)^, respectively (Table 2). Piglets received a volume of 1·5 ml of each treatment during the first period (0–7 d of age) except for creatine treatments where the volume was 3·0 ml. During the second period (8–14 d of age), piglets received 3·0 ml of each treatment except for creatine treatments (6 ml). Finally, during the third period (15–21 d of age), piglets received 4·5 ml of each treatment except for creatine treatments (9·0 ml). For oral boluses containing creatine, the complete delivery was insured by continuous mixing during administration.

**Table 2.** Amounts of each nutrient administered orally to piglets according to age

Age* (d)	Betaine† (g/d)	Creatine‡ (g/d)	Choline§ (mg/d)	Pyridoxine|| (mg/d)
0–7	0·1	0·6	140	0·4
8–14	0·2	1·2	280	0·8
15–21	0·3	1·8	560	1·2

* These daily allowances were adjusted for expected body weights of 2, 4 and 6 kg at 0–7, 8–15 and 15–21 d of age. The actual average body weights were 2·2, 4·3 and 6·5 kg.

† Based on the average supplementation per kg body weight in growing–finishing pigs according to Eklund *et al.*^(^^15^^)^ and adjusted for expected body weights of piglets.

‡ Based on total daily accretion per kg body weight in piglets according to Brosnan *et al.*^(^^16^^)^ and adjusted for expected body weights of piglets.

§ Based on the total amount of choline provided by sows’ milk per kg body weight in piglets according to Donovan *et al.*^(^^17^^)^ and adjusted for expected body weights of piglets.

|| Based on optimal dietary provision per kg body weight in piglets after weaning according to Matte *et al.*^(^^18^^)^ and adjusted for expected body weights of piglets.

### Sampling and analytical measurements

Blood samples were collected in EDTA-containing tubes (7 ml; Becton Dickinson and Co.). The tubes were placed on ice until all samples were collected. Then, the tubes were mixed gently for 5 min before determination of packed cell volume as described by Matte *et al.*^(^^13^^)^ The remaining blood was centrifuged during 12 min at 4°C (1800 ***g***). Plasma and erythrocytes were separated and frozen at −20°C until analysed.

Concentration of total pHcy and cysteine were measured by HPLC according to the method validated by Simard *et al.*^(^^4^^)^. Blood plasma concentrations of creatine, choline and sarcosine were determined by commercial colorimetric kits (Biovision Research Products K615-100, K636-100 and K635-100, respectively) according to the manufacturer's instructions. Validation tests for measurement of creatine, choline and sarcosine in blood plasma showed satisfactory parallelism (CV < 9·7 %) and recovery test (98·5–102·5 %). Intra- and interassay CV were lower than 6·1 and 13·0 %, respectively. Pyridoxal-5-phosphate (P-5-P) was determined in erythrocytes and milk using the sample preparation and method adapted for erythrocytes by Matte *et al.*^(^^19^^)^ from Srivastava & Beutler^(^^20^^)^.

### Statistical analyses

Data were analysed according to a randomised arrangement of treatments in blocks (*n* = 16–17, except for plasma concentrations of choline, creatine and sarcosine where *n* = 7–9) and piglets within litter were considered as experimental units. Main effects of treatments (*n* = 8) and ages at sampling (*n* = 5) along with the interaction treatment × age were analysed using the repeated option of the SAS procedure for mixed models (SAS Institute Inc.)^(^^21^^)^. Several covariance structures were compared and the Ante-dependence covariable (ANTE) structure was retained as it yielded the best-fit statistical value. Differences were considered to be significant at *P* < 0·05 and a tendency at 0·05 ≤ *P* ≤ 0·10. A Tukey–Kramer correction was applied for all pairwise comparisons when main effects tended to be or were significant. All results are expressed as least-squares means with standard errors of the mean.

## Results and discussion

### Body condition of sows and growth of piglets

Average body weight and backfat thickness of sows were, respectively, 205·8 (sem 3·1) kg and 16·0 (sem 0·8) mm at mating, 251·0 (sem 3·9) kg and 16·9 (sem 1·0) mm on day 1 after parturition, and 244·0 (sem 4·1) kg and 15·1 (sem 1·0) mm at weaning. Measurements from nine piglets were missing at some stages of the lactation period due to weakness or crushing by their mother but these losses were not related to treatments. In one litter, treatments 1 and 8 were missing for the whole experimental period.

Growth performance of piglets was monitored during the lactation period. No treatment effect was observed on body weight (*P* > 0·54) or average daily gain (*P* > 0·13) of piglets during the lactation period. Average body weights were 1·71 (sem 0·01) kg at birth and 1·75 (sem 0·02), 3·17 (sem 0·04), 5·34 (sem 0·08) and 7·53 (sem 0·12) at 1, 7, 14 and 21 d of age, respectively whereas average daily gain was 0·18 (sem 0·01), 0·24 (sem 0·01) and 0·26 (sem 0·01) kg from 1 to 7, 7 to 14 and 14 to 21 d of age, respectively. Although growth of suckling piglets was not influenced by the different treatments, average daily gain and body weight of these animals during lactation reflect only partially the overall growth performance because no data are available on DM intake and feed conversion from milk consumption for each piglet.

### Age and treatment responses of plasma homocysteine and cysteine

**Fig. 1.**
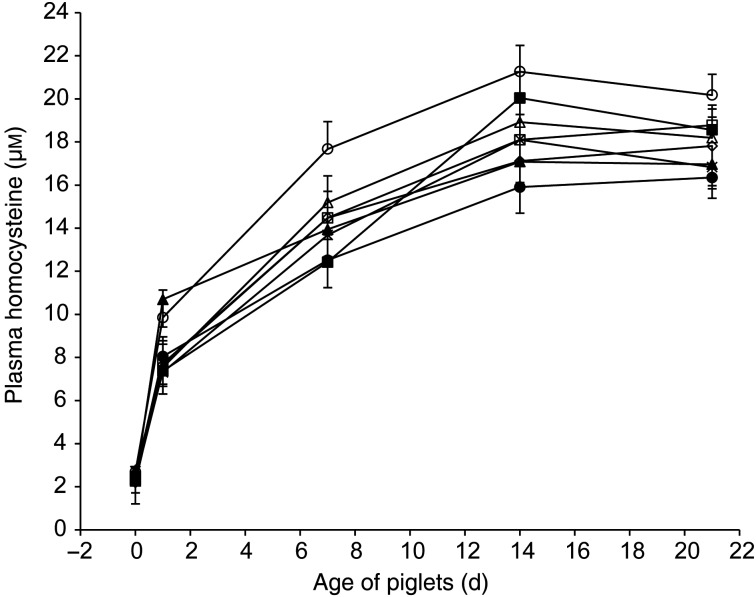
Plasma concentrations of homocysteine according to treatment and age of piglets: ○, control (treatment 1); □, betaine (treatment 2); ∆, choline (treatment 3); ×, creatine (treatment 4); ◦, pyridoxine (treatment 5); ■, betaine and pyridoxine (treatment 6); ▲, choline and creatine (treatment 7); ●, all nutrients (treatment 8). Values are least-square means (*n* 16, 17, 17, 17, 17, 17, 17 and 16 piglets for treatments 1, 2, 3, 4, 5, 6, 7 and 8), with standard errors represented by vertical bars. There was an age effect (*P* < 0·01). There was a tendency for a treatment effect (*P* = 0·09). Pairwise comparison (Tukey–Kramer test) between treatments 1 and 8 was significant (*P* = 0·03).

Overall plasma concentration of pHcy was very low at birth (2·48 (sem 0·06) μm), increased sharply to 8·27 (sem 0·44) μm within 24 h, and to 14·30 (sem 0·45) and 18·32 (sem 0·43) μm at 7 and 14 d of age, respectively, and plateaued during the last week of lactation with a value of 17·96 (sem 0·34) μm at 21 d of age (age effect; *P* < 0·01) (Fig. 1). This main age effect is consistent with that reported, under similar conditions, in other studies^(^^3^^–^^5^^)^. According to Simard *et al.*^(^^4^^)^, such high concentrations of pHcy were probably due to piglet metabolism because Hcy is undetectable in milk. Further analyses of the present milk samples with mass spectrophotometric detection confirmed the absence of Hcy in milk from all sows whereas methionine corresponded to 1·2 (sem 0·06) g/l.

In humans, pHcy concentrations increase from 20 to 40 d of age in breastfed infants (6·7–9·1 µm) but not in formula-fed infants^(^^22^^)^. Nutritional factors have been raised to explain this phenomenon, in particular, the differences in B-vitamin intakes which were three (vitamin B_6_) to five times (vitamin B_12_, vitamin B_2_ and folates) higher in formula-fed than in breastfed infants. Such difference was probably amplified by a depressed B-vitamin status in lactating women during this period^(^^22^^)^. In the present experiment, it is unlikely that milk folates or vitamin B_12_ were limiting because sow diets during gestation and lactation were supplemented at levels of 10 mg/kg of folic acid and 150 µg/kg of cyanocobalamin. Such supplements in sow feed are known to maximise milk transfer of folates^(^^23^^,^^24^^)^ and vitamin B_12_^(^^5^^)^ to piglets during lactation and reduce the postnatal rise of pHcy in piglets, probably via the cellular methionine synthase remethylation pathway as stated by Simard *et al.*^(^^4^^)^. Other metabolic pathways for Hcy synthesis or disposal were addressed with the different nutrients used in the present experiment and concentrations of pHcy tended to be lower (*P* = 0·09) in treated than in control piglets (Fig. 1). However, the highest and sole pairwise significant decrease (23 %) was observed between control and treatment 8 (14·33 (sem 0·70) *v.* 11·05 (sem 0·70) μm, respectively; *P* = 0·03). None of the other pairwise comparisons between control (treatment 1) and each nutrient given alone (treatments 2, 3, 4 and 5) or in combination of two (treatments 6 and 7) reached significance (*P* > 0·15). There was no treatment × age interaction (*P* > 0·11). As mentioned earlier, two approaches, besides the methionine synthase remethylation pathway, were chosen to prevent the acute increase of pHcy in suckling piglets. The first one, using dietary betaine through the betaine-Hcy-methyltransferase pathway and pyridoxine through vitamin B_6_-dependent trans-sulfuration, aimed to enhance the disposal of Hcy while the second one provided preformed methylated nutrients (creatine and choline) to limit the need for methyl groups and, then, prevent Hcy formation. It seems that combining both approaches was necessary to attenuate the rapid rise of homocysteinaemia in neonatal piglets. However, although the response to treatment 8 represented a decrease of pHcy of 23 %, the lowest average concentration achieved from 1 to 21 d of age, at 13·1 µm, was similar to the mean comparable value of 13·2 µm observed during the suckling period after one intramuscular injection of vitamin B_12_ administered at 1 d of age to piglets^(^^5^^)^. It appears that supplements of vitamin B_12_ given to piglets by injection, which had probably activated the cellular methionine synthase remethylation pathway for disposal of Hcy, were as efficient as the present dual approaches pursued with the cocktail of nutrients (treatment 8) to reduce pHcy increase during the suckling period. Additional studies are needed to know if a synergy among all those approaches (present cocktail of nutrients and intramuscular injections of vitamin B_12_ used by Audet *et al.*^(^^5^^)^) can enhance the clearance rate of Hcy in suckling piglets because even these lowest concentrations of pHcy in suckling piglets remain substantially higher than typical levels in other species (<10 µm), as stated by Simard *et al.*^(^^4^^)^.

As for pHcy, plasma cysteine increased with age (*P* < 0·01) from 65·5 (sem 1·2) μm at birth to 99·3 (sem 1·7), 159·8 (sem 2·4), 198·3 (sem 2·2) and 209·4 (sem 2·1) μm on days 1, 7, 14 and 21 of age but it was not affected by treatments (*P* > 0·67; Table 3). This age response is similar to that reported by Ballance & House^(^^3^^)^ and Simard *et al.*^(^^4^^)^. In this last study, an increase of vitamin B_6_-dependent enzyme activities in the trans-sulfuration pathway (cystathione-*β* synthase and cystathionine-*γ*-lyase) was also reported between birth and 26 d of age. Nevertheless, in spite of these age-related enzymic changes, it cannot be ruled out that the consumption of cystine from sows’ milk^(^^25^^)^ may be a main contributing factor to cysteine homeostasis in piglets during the suckling period and may have masked plasma cysteine responses to treatments with vitamin B_6_ supplements.

**Table 3.** Plasma concentrations of cysteine, choline and sarcosine in piglets during the first 21 d of age according to treatments (Least-square mean values with their pooled standard errors)

	Plasma metabolite		
Nutrient and treatment no.	Cysteine (μm)* §	Sarcosine (μm)†||	Choline (μm)‡ ¶
Control (treatment 1)	152·83	16·26	13·44^a,b^
Betaine (treatment 2)	143·69	17·54	12·10^a^
Choline (treatment 3)	144·70	17·37	13·56^a,b^
Creatine (treatment 4)	147·73	15·92	13·11^a,b^
Pyridoxine (treatment 5)	142·38	17·60	11·84^a^
Betaine + pyridoxine (treatment 6)	145·11	16·47	13·67^a,b^
Choline + creatine (treatment 7)	148·24	15·10	14·06^a,b^
All nutrients (treatment 8)	147·13	15·65	15·84^b^
sem	<3·95	<0·70	<0·80

^a,b^ Mean values within a column with unlike superscript letters were significantly different (*P* < 0·05).

* Values are least-square means of 16, 17, 17, 17, 17, 17, 17 and 16 piglets for treatments 1, 2, 3, 4, 5, 6, 7 and 8.

† Values are least-square means of nine piglets for each treatment.

‡ Values are least-square means of seven piglets for each treatment.

§ Age effect (see text; *P* < 0·01).

|| Tendency for a treatment effect (*P* = 0·09) but no pairwise comparison (Tukey–Kramer test) was significant (*P* > 0·17).

¶ Treatment effect (*P* = 0·01).

### *Creatine, choline and vitamin B*_*6*_ *homeostasis in piglets during the suckling period*

Except for betaine, blood plasma concentrations of each studied nutrient in the present experiment were monitored during the experimental period.

**Fig. 2.**
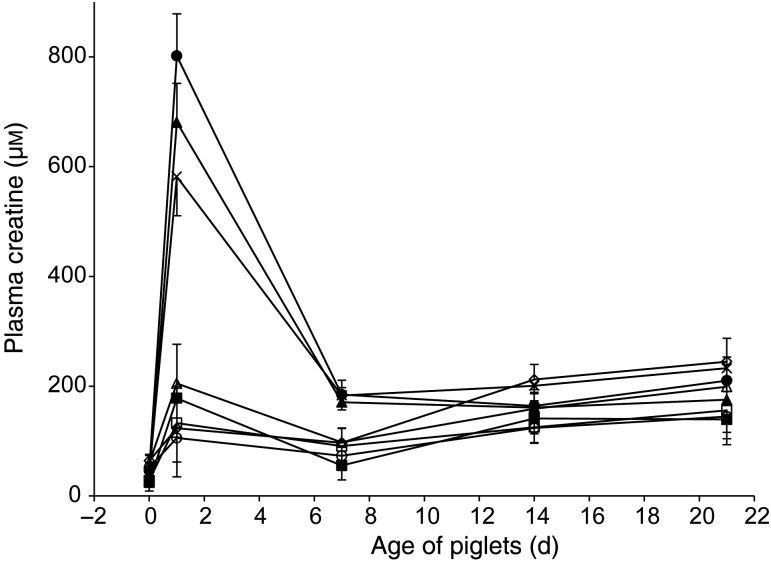
Plasma concentrations of creatine according to treatment and age of piglets: ○, control (treatment 1); □, betaine (treatment 2); ∆, choline (treatment 3); ×, creatine (treatment 4); ◦, pyridoxine (treatment 5); ■, betaine and pyridoxine (treatment 6); ▲, choline and creatine (treatment 7); ●, all nutrients (treatment 8). Values are least-square means of seven piglets for each treatment, with standard errors represented by vertical bars. There was an age effect (*P* < 0·01). There was a treatment × age interaction (*P* < 0·01).

As mentioned earlier, both creatine and choline were provided to reduce the demand for methyl groups and eventually the formation of Hcy. For choline, it has a dual role on Hcy metabolism because betaine, produced by oxidation of choline, could also promote remethylation of Hcy. Due to technical reasons, it was not possible to measure the plasma concentration of betaine in the present experiment. Plasma concentrations of creatine in creatine-supplemented treatments (4, 7 and 8) peaked at 1 d of age and decreased thereafter to be no longer different from in other treatments from 14 to 21 d of age (interaction treatment × age; *P* < 0·01) (Fig. 2). Such response suggests that the provision of exogenous preformed creatine exceeds the metabolic utilisation of this nutrient in piglets during their first days of life but not later in lactation. Nevertheless, it cannot be ruled out that this response could also be related to the efficiency of excretion of creatine in creatinine influenced by the postnatal development of renal function in piglets during the first weeks of life^(^^26^^,^^27^^)^. In the case of plasma choline, for all treatments, concentrations increased (age effect; *P* < 0·01) from 9·88 (sem 0·53) and 10·97 (sem 0·52) μm at birth and 1 d of age, respectively, to 15·50 (sem 0·67), 15·94 (sem 0·43) and 14·97 (sem 0·40) μm at 7, 14 and 21 d of age. A treatment effect on plasma choline was detected (*P* = 0·01) with the lowest concentrations for oral supplementations of betaine and pyridoxine (treatments 2 and 5) and the highest one with the cocktail of all treatments (treatment 8) (Table 3). It is noteworthy that, in spite of this treatment effect, none of the pairwise comparisons between individual or combined nutrients *v*. the control treatment was significant (*P* > 0·31). There was no treatment × age interaction (*P* > 0·94) on plasma choline concentrations.

Although not specifically measured in the actual study, creatine and choline are present in sows’ milk but their provisions are not considered sufficient to meet piglet requirements^(^^9^^,^^17^^)^. The present supplementations aimed to approximately double the estimated^(^^9^^,^^17^^)^ total daily provision of creatine and choline to piglets. They can be both synthesised by the animal, a process involving methylation brought by the conversion of *S*-adenosyl-methionine to *S*-adenosyl-Hcy. Three methyl groups are required for synthesis of phosphatidylcholine and one for creatine^(^^10^^)^. Phosphatidylcholine can be also derived from choline through the Kennedy pathway^(^^10^^)^. For creatine, the major proportion of accretion in piglets comes from *de novo* synthesis^(^^9^^)^ which, in turn, accounted for the major part (approximately 77 %) of the daily requirement for labile methyl groups^(^^9^^,^^28^^)^. In terms of metabolic balance of methyl groups, if the present single (treatments 3 and 4) or combined (treatments 7 and 8) provisions of choline and creatine reduced the demand of methyl groups over their endogenous synthesis, this could have triggered metabolic mechanisms for the outflow of methyl groups such as sarcosine production and degradation^(^^29^^)^. In this respect, plasma concentrations of sarcosine were measured in the present study. They increased from birth to 1 d of age (13·29 (sem 0·06) to 16·26 (sem 0·08) μm, respectively), reached a maximum at 7 d of age (19·73 (sem 0·05) μm) and then declined and remained stable from 14 to 21 d of age (16·92 (sem 0·03) and 16·25 (sem 0·04) μm, respectively) (age effect; *P* < 0·01). There was a tendency for a treatment effect (*P* = 0·09) but none of the pairwise comparison among treatments was significant (*P* > 0·17; Table 3). These results suggest, therefore, that none of the present treatments had generated an excess of methyl groups in piglets.

**Fig. 3.**
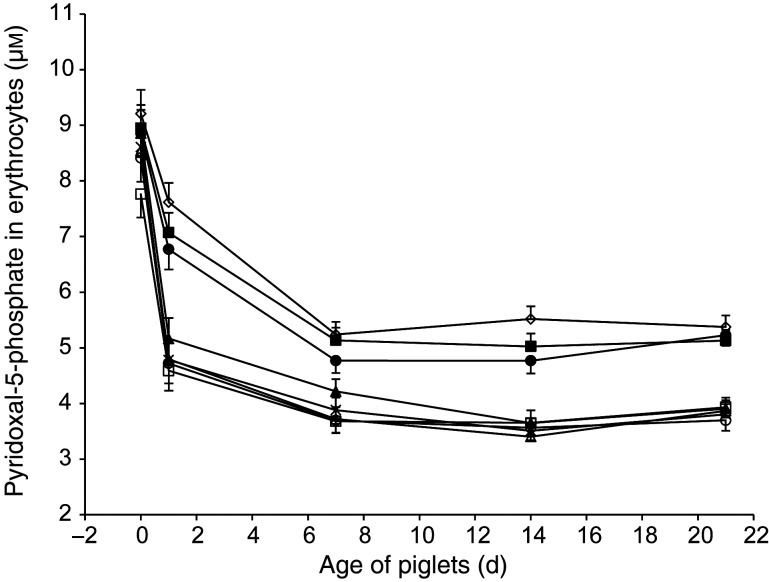
Pyridoxal-5-phosphate concentrations in erythrocytes according to treatment and age of piglets: ○, control (treatment 1); □, betaine (treatment 2); ∆, choline (treatment 3); ×, creatine (treatment 4); ◦, pyridoxine (treatment 5); ■, betaine and pyridoxine (treatment 6); ▲, choline and creatine (treatment 7); ●, all nutrients (treatment 8). Values are least-square means (*n* 16, 17, 17, 17, 17, 17, 17 and 16 piglets for treatments 1, 2, 3, 4, 5, 6, 7 and 8), with standard errors represented by vertical bars. There was an age effect (*P* < 0·01).There was a treatment × age interaction (*P* = 0·02).

Another route for the disposal of Hcy is catabolism through the trans-sulfuration pathway where the vitamin B_6_-dependent cystathionine β-synthase and cystathionine-*γ*-lyase produce cystathionine and then cysteine and glutathione^(^^30^^)^. In the present experiment, the fate of pyridoxine in the different treatments was assessed using P-5-P in erythrocytes as the indicator according to Matte *et al.*^(^^18^^)^. Globally, for all treatments, P-5-P concentrations decreased by 50 % from day 0 to day 7 of lactation and remained stable thereafter until day 21, though at greater concentrations with vitamin B_6_-supplemented treatments 5, 6 and 8 than with others (treatment × age interaction; *P* = 0·02) (Fig. 3). This response on P-5-P along with the lack of treatment 5 (vitamin B_6_ alone) effect on Hcy contrasts with Zhang *et al.*^(^^31^^)^ who showed that the contribution of the vitamin B_6_-dependent trans-sulfuration pathway is crucial for the variations of homocysteinaemia in a vitamin B_6_ depletion–repletion model with weaned piglets. These last authors observed the maximum effect of dietary vitamin B_6_ on pyridoxal -phosphate and pHcy concentrations with an intake of 2·3 mg/d. In the present experiment, during the last week of lactation, the total daily intake of vitamin B_6_ for the suckling piglets corresponded to the oral supplement (1·2 mg) along with the vitamin B_6_ intake from milk which can be estimated at 1·4 mg using an average daily milk consumption of 0·8 litres and a vitamin B_6_ concentration in sows’ milk of 1·7 (sem 0·06) mg/l. Thus, the total daily intake of vitamin B_6_ in vitamin B_6_-supplemented treatments 5, 6 and 8 was approximately 2·6 mg, an amount comparable with the dietary level used by Zhang *et al.*^(^^31^^)^ for a maximal reduction of pHcy. However, in Zhang *et al.*^(^^31^^)^, the basal diet was not supplemented with betaine or creatine but choline was provided in their vitamin premix for a daily intake of approximately 800 mg. In the present experiment, the combination of vitamin B_6_ and choline (560 mg per d) supplementations was present only in treatment 8 (all supplemented nutrients) where pHcy was significantly decreased as compared with treatment 1 (control). Therefore, it appears that the synergy between these two nutrients may be non-negligible contributors to the pHcy-lowering effect observed in treatment 8. In fact, as plasma sarcosine results probably indicate that no excess of methyl groups was produced with the present supply of nutrients reducing the demand of methyl groups it cannot be excluded that the present provisions of choline and possibly creatine were not optimal for a maximal lowering effect on the development of homocysteinaemia in suckling piglets.

### Conclusion

Concentrations of pHcy were reduced by 23 % with the combination of the nutrients betaine, pyridoxine, choline and creatine supplied directly to suckling piglets during lactation. Nevertheless, minimum values of pHcy remained high (>13 µm) as compared with normal concentrations in other species (<10 µm) and the question remains as to whether a greater reduction of this intermediary amino acid is achievable and, eventually, worthwhile for the growth and immune function of piglets.
